# Supplementary material for: Effectiveness of inactivated COVID-19 vaccines among older adults in Shanghai: retrospective cohort study
Source: Nat Commun. 2023 Apr 10;14:2009. doi: 10.1038/s41467-023-37673-9 (PMC10086022; doi:10.1038/s41467-023-37673-9)
Supplement: Supplementary file 1 — Supplementary Information [file 41467_2023_37673_MOESM1_ESM.pdf]

Table S1 Baseline Demographic Characteristics of Cohort 1 and Cohort 2 (Controls Selection with Sampling Without Replacement)

	Cohort 1			Cohort 2		
	**Unvaccinated group (N = 2,903,292)**	**Vaccinated group (N = 2,903,292)**	**SMD**	**Fully vaccinated group (N = 1,857,411)**	**Booster vaccinated group (N = 1,847,411)**	**SMD**
Median age [IQR] — years-old	67.0 [63.0, 72.0]	67.0 [63.0, 72.0]	<0.01	67.0 [64.0, 71.0]	67.0 [64.0, 71.0]	<0.01
Age group — no. (%)						
60 – 69 years-old	1,895,552 (65.3)	1,895,552 (65.3)	<0.01	1,275,493 (69.0)	1,275,493 (69.0)	<0.01
70 – 79 years-old	819,954 (28.2)	819,954 (28.2)		508,036 (27.5)	508,036 (27.5)	
80 – 89 years-old	175,786 (6.1)	175,786 (6.1)		61,787 (3.3)	61,787 (3.3)	
90 years-old and above	12,000 (0.4)	12,000 (0.4)		2,095 (0.1)	2,095 (0.1)	
Sex — no. (%)						
Male	1,472,713 (50.7)	1,472,713 (50.7)	<0.01	920,611 (49.8)	920,611 (49.8)	<0.01
Female	1,430,579 (49.3)	1,430,579 (49.3)		926,800 (50.2)	926,800 (50.2)	
Chronic disease — no. (%)						
0	1,669,719 (57.5)	1,669,719 (57.5)	<0.01	1,160,279 (62.8)	1160,279 (62.8)	<0.01
≥1	1,233,573 (42.5)	1,233,573 (42.5)		687,132 (37.2)	687,132 (37.2)	
Cancer	233,159 (8.0)	130,952 (4.5)	0.04	73,177 (4.0)	66,913 (3.6)	0.02
Hypertension	981,600 (33.8)	1,041,885 (35.9)	0.04	578,198 (31.3)	580,222 (31.4)	<0.01
Diabetes	355,445 (12.2)	316,394 (10.9)	0.04	174,227 (9.4)	176,418 (9.5)	<0.01
Vaccination status — no. (%)						
Unvaccinated	2,903,292 (100.0)	-	-	-	-	-
Partial vaccination	-	475,702 (16.4)		-	-	
Full vaccination	-	1,360,306 (46.9)		1,847,411 (100.0)	-	
Booster vaccination	-	1,067,284 (36.8)		-	1,847,411 (100.0)	
Time interval between 1st dose and 2nd dose — no. (%)						
Median [IQR] — days	-	-		23.0 [21.0, 28.0]	23.0 [21.0, 26.0]	<0.01
21 − 56 days	-	-		1,832,049 (99.2)	1,832,049 (99.2)	<0.01
57 – 180 days	-	-		14,951 (0.8)	14,951 (0.8)	
181 days and above	-	-		411 (0.0)	411 (0.0)	
Time interval since completion of 2nd dose — no. (%)						
Median [IQR] — days	-	-		202.0 [190.0, 224.0]	212.0 [196.0, 238.0]	0.32
181 − 270 days	-	-		1,714,818 (92.8)	1,714,818 (92.8)	<0.01
271 − 360	-	-		132,165 (7.2)	132,165 (7.2)	
361 days and above	-	-		428 (0.0)	428 (0.0)	

SMD：standardized mean difference.

Table S2 Sensitivity Analyses of Vaccine Effectiveness of Inactivated Covid-19 Vaccine against Covid-19 Outcomes

	Cohort 1			Cohort 2		
	**Documented SARS-CoV-2 Infection**	**Severe/critical Covid-19**	**Covid-19 related death**	**Documented SARS-CoV-2 Infection**	**Severe/critical Covid-19**	**Covid-19 related death**
**Negative binomial regression**						
Overall	23.8 (22.8, 24.8)	86.4 (81.7, 90.0)	93.3 (86.9, 96.8)	15.8 (14.1, 17.4)	57.2 (22.2, 76.9)	81.7 (-16.2, 98.2)
Age group						
60 – 69 years-old	21.8 (20.5, 23.1)	92.3 (86.4, 95.8)	96.8 (83.4, 99.6)	14.6 (12.7, 16.5)	39.6 (-95.3, 82.0)	100 (NA)
70 – 79 years-old	25.6 (23.6, 27.5)	79.9 (68.9, 87.2)	89.6 (73.4, 96.3)	19.9 (16.8, 22.8)	62.9 (-9.2, 88.0)	43.3 (NA)
80 – 89 years-old	38.0 (33.9, 41.8)	86.4 (78.3, 91.9)	94.2 (77.2, 98.9)	4.6 (-7.3, 15.1)	70.6 (4.5, 91.6)	100 (NA)
90 years-old and above	50.3 (34.8, 62.4)	73.5 (26.9, 92.4)	92.5 (-52.0, 100.0)	3.8 (-68.9, 45.3)	None*	None*
Sex — no.						
Male	22.4 (20.9, 23.9)	86.4 (76.3, 92.4)	95.7 (83.0, 99.2)	19.0 (16.7, 21.1)	67.1 (-32.4, 93.0)	84.7 (NA)
Female	25.2 (23.7, 26.6)	86.5 (80.8, 90.6)	91.9 (82.4, 96.6)	12.5 (10.1, 14.8)	55.1 (11.5, 77.6)	78.5 (-195.9, 99.3)
Chronic disease						
0	20.6 (19.1, 2.02)	88.0 (81.2, 92.5)	97.2 (89.5, 99.4)	12.2 (10.1, 14.3)	57.1 (0.4, 82.3)	85.7 (-62.4, 99.4)
⩾1	28.3 (26.8, 29.8)	84.9 (78.0, 89.8)	87.4 (71.7, 94.8)	21.9 (19.3, 24.4)	57.7 (5.5, 81.6)	70.6 (-661.0, 99.6)
Time interval between 1st dose and 2nd dose (days)						
21 − 56 days	-	-	-	15.8 (14.2, 17.4)	55.5 (18.5, 76.0)	81.7 (-16.2, 98.2)
57 – 180 days	-	-	-	3.8 (-21.7, 23.9)	100 (NA)	-
181 days and above	-	-	-	-	-	-
Time interval since completion of 2nd dose (days)						
181 − 270	-	-	-	15.5 (13.8, 17.2)	54.5 (16.4, 75.6)	81.8 (-15.9, 98.2)
271 − 360	-	-	-	18.4 (12.2, 24.2)	100 (NA)	-
361 and above	-	-	-	100.0 (13.8, 17.2)	-	-
**Resampling matched controls without replacement**						
Overall	27.7 (26.7, 28.8)	85.8 (81.9, 88.8)	91.0 (84.4, 94.8)	14.4 (12.7, 16.1)	66.1 (43.7, 79.6)	80.1 (9.3, 95.6)
Age group						
60 – 69 years-old	26.8 (25.4, 28.1)	92.7 (87.7, 95.7)	97.9 (84.9, 99.7)	14.0 (12.0, 15.9)	60.5 (-1.9, 84.7)	None*
70 – 79 years-old	27.4 (25.5, 29.4)	79.0 (70.8, 84.9)	83.7 (68.1, 91.7)	16.2 (12.8, 19.3)	80.1 (52.1, 91.7)	83.5 (-36.9, 98.0)
80 – 89 years-old	38.1 (34.1, 41.9)	86.1 (76.2, 91.9)	95.3 (80.8, 98.9)	9.9 (-1.5, 20.0)	61.5 (-7.9, 86.3)	None*
90 years-old and above	48.3 (31.7, 60.9)	77.6 (21.5, 93.6)	49.7 (-455.0, 95.4)	None*	None*	None*
Sex						
Male	25.2 (23.6, 26.8)	85.6 (77.7, 90.7)	97.9 (84.9, 99.7)	16.5 (14.2, 18.8)	50.0 (-46.4, 82.9)	None*
Female	30.2 (28.7, 31.6)	85.9 (81.1, 89.4)	87.9 (78.4, 93.2)	12.2 (9.8, 14.6)	69.4 (45.4, 82.8)	88.9 (12.5, 98.6)
Chronic disease — no.						
0	24.9 (23.3, 26.4)	88.5 (82.9, 92.3)	92.2 (82.2, 96.6)	11.8 (9.6, 13.9)	76.1 (41.6, 90.2)	None*
⩾1	30.8 (29.3, 32.3)	83.8 (78.0, 88.0)	89.8 (78.8, 95.1)	19 (16.3, 21.6)	58.8 (23.2, 77.9)	60.5 (-103.7, 92.3)
Time interval between 1st dose and 2nd dose (days)						
21 − 56 days	-	-	-	14.5 (12.8, 16.1)	66.1 (43.7, 79.6)	80.1 (9.3, 95.6)
57 – 180 days	-	-	-	8.6 (-15.9, 28.0)	-	-
181 days and above	-	-	-	-	-	-
Time interval since completion of 2nd dose (days)						
181 − 270	-	-	-	14.3 (12.6, 16)	64.9 (41.6, 78.9)	80.1 (9.3, 95.6)
271 − 360	-	-	-	17.4 (8, 25.9)	None*	-
361 and above	-	-	-	-	-	-
**Including matched pairs with follow-up time within 7 days**						
0-7 days	97.8 (91.2, 99.5)	None*	None*	90.4 (80.1, 95.3)	None*	-
8-60 days	41.7 (32.8, 49.5)	57.2 (-65.5, 88.9)	None*	25.3 (21.6, 28.8)	69.1 (5.2, 89.9)	None*
61-180 days	7.6 (0.9, 13.9)	None*	90.0 (21.8, 98.7)	14.1 (12.3, 15.8)	57.6 (30.2, 74.2)	74.9 (-18.3, 94.7)
⩾181 days	25.0 (23.9, 26.0)	86.2 (82.5, 89.1)	93.2 (87.9, 96.2)	55.1 (17.4, 75.6)	-	-

* No evidence of protection based on a negative or very small positive point estimate and wide CIs
NA: not available

Table S3 Adjusted Vaccine Effectiveness of Inactivated Covid-19 Vaccine against Covid-19 Outcomes by Vaccine Brand

	Cohort 1			Cohort 2		
	**Documented SARS-CoV-2 Infection**	**Severe/critical Covid-19**	**Covid-19 related death**	**Documented SARS-CoV-2 Infection**	**Severe/critical Covid-19**	**Covid-19 related death**
**By vaccine brand**						
Unvaccinated	Reference level	Reference level	Reference level	-	-	-
One dose Sinovac-CorV	None*	None*	None*	-	-	-
One dose BIBP-CorV	None*	None*	None*	-	-	-
One dose WIBP-CorV	None*	None*	None*	-	-	-
One dose other inactive vaccine	None*	None*	None*	-	-	-
Two-dose Sinovac-CorV	None*	65.5 (50.9, 75.7)	80.4 (55.8, 91.3)	Reference level	Reference level	Reference level
Two-dose BIBP-CorV	None*	76.4 (61.8, 85.4)	82.7 (53.4, 93.6)	None*	None*	None*
Two-dose WIBP-CorV	None*	None*	None*	14.3 (0.1, 26.5)	None*	None*
Mixed two doses inactive vaccine	None*	None*	None*	None*	None*	None*
Three-dose Sinovac	51.7 (50.6, 52.8)	94.9 (91.0, 97.1)	None*	18.0 (15.8, 20.0)	51.4 (12.0, 73.2)	None*
Three-dose BIBP-CorV	50.6 (49.3, 51.8)	95.5 (90.9, 97.7)	98.2 (86.9, 99.7)	16.8 (14.4, 19.1)	62.8 (22.5, 82.2)	None*
Three-dose WIBP-CorV	66.7 (59.9, 72.3)	None*	None*	42.2 (31.9, 51.0)	None*	None*
Mixed three doses inactive vaccine	26.6 (20.9, 31.9)	None*	None*	None*	None*	None*

* No evidence of protection based on a negative or very small positive point estimate and wide CIs
