# Supplementary material for: Effectiveness of inactivated COVID-19 vaccines among older adults in Shanghai: retrospective cohort study
Source: Nat Commun. 2023 Apr 10;14:2009. doi: 10.1038/s41467-023-37673-9 (PMC10086022; doi:10.1038/s41467-023-37673-9)
Supplement: Supplementary file 3 — Reporting Summary [file 41467_2023_37673_MOESM3_ESM.pdf]

nature portfolio

Corresponding author(s): Dr. Weibing Wang

Last updated by author(s): Mar 14, 2023

Reporting Summary

Nature Portfolio wishes to improve the reproducibility of the work that we publish. This form provides structure for consistency and transparency in reporting. For further information on Nature Portfolio policies, see our Editorial Policies and the Editorial Policy Checklist.

Statistics

For all statistical analyses, confirm that the following items are present in the figure legend, table legend, main text, or Methods section.

n/a	Confirmed	
☐	☒	The exact sample size (*n*) for each experimental group/condition, given as a discrete number and unit of measurement
☐	☒	A statement on whether measurements were taken from distinct samples or whether the same sample was measured repeatedly
☐	☒	The statistical test(s) used AND whether they are one- or two-sided<br>*Only common tests should be described solely by name; describe more complex techniques in the Methods section.*
☐	☒	A description of all covariates tested
☐	☒	A description of any assumptions or corrections, such as tests of normality and adjustment for multiple comparisons
☐	☒	A full description of the statistical parameters including central tendency (e.g. means) or other basic estimates (e.g. regression coefficient) AND variation (e.g. standard deviation) or associated estimates of uncertainty (e.g. confidence intervals)
☐	☒	For null hypothesis testing, the test statistic (e.g. *F*, *t*, *r*) with confidence intervals, effect sizes, degrees of freedom and *P* value noted<br>*Give P values as exact values whenever suitable.*
☒	☐	For Bayesian analysis, information on the choice of priors and Markov chain Monte Carlo settings
☒	☐	For hierarchical and complex designs, identification of the appropriate level for tests and full reporting of outcomes
☒	☐	Estimates of effect sizes (e.g. Cohen's *d*, Pearson's *r*), indicating how they were calculated

Our web collection on statistics for biologists contains articles on many of the points above.

Software and code

Policy information about availability of computer code

Data collection	No software was used for data collection
Data analysis	The data were analyzed using survival package (3.3-1), and survminer package (0.4.9) in R version 4.1.3, and no custom codes were developed.

For manuscripts utilizing custom algorithms or software that are central to the research but not yet described in published literature, software must be made available to editors and reviewers. We strongly encourage code deposition in a community repository (e.g. GitHub). See the Nature Portfolio guidelines for submitting code & software for further information.

Data

Policy information about availability of data

All manuscripts must include a data availability statement. This statement should provide the following information, where applicable:

- Accession codes, unique identifiers, or web links for publicly available datasets
- A description of any restrictions on data availability
- For clinical datasets or third party data, please ensure that the statement adheres to our policy

The dataset from this study is held in coded form at Shanghai Center for Disease Control and Prevention. While legal data sharing agreements between Shanghai CDC and its superior departments in charge (e.g., Health Commission or local government) prohibit Shanghai CDC from making the dataset publicly available. Access may be granted to those who through a request with specific data needs, analysis plans, and dissemination plans to Zhuoying Huang (e-mail:

huangzhuoying@scdc.sh.cn), Dr. Xiaodong Sun (e-mail: sunxiaodong@scdc.sh.cn), and Dr. Weibing Wang (e-mail: wwb@fudan.edu.cn). The authors will give feedback within 30 days. However, individual identification information may not be available for public use.

Human research participants

Policy information about studies involving human research participants and Sex and Gender in Research.

Reporting on sex and gender	Sex were self reported. We have reported our findings by sex.
Population characteristics	All eligible participants in Shanghai were included in the study. In cohort 1, the median age was 67 years, 49.4% were female, and 39.4% had ≥1 chronic disease. In cohort 2, the median age was 67 years, 50.7% were female, and 37.7% had ≥ 1 chronic disease.
Recruitment	Participants were not recruited. Relevant participant data were extracted retrospectively from Shanghai Group Immunization System, National Immunization Program Information System, and the National Notifiable Diseases Registry System.
Ethics oversight	The study was approved by the Ethical Review Committee in the Shanghai Center for Disease Control and Prevention (approval number: 2022-20).

Note that full information on the approval of the study protocol must also be provided in the manuscript.

Field-specific reporting

Please select the one below that is the best fit for your research. If you are not sure, read the appropriate sections before making your selection.

☒ Life sciences ☐ Behavioural & social sciences ☐ Ecological, evolutionary & environmental sciences

For a reference copy of the document with all sections, see nature.com/documents/nr-reporting-summary-flat.pdf

Life sciences study design

All studies must disclose on these points even when the disclosure is negative.

Sample size	This is an observational, retrospective, matched cohort study in a community-dwelling population aged 60 years and older in Shanghai. Almost all inactivated Covid-19 vaccine recipients aged 60 years and older in Shanghai were included, thus no sample-size calculation was performed.
Data exclusions	Potential subjects with a previous documented SARS-CoV-2 infection, or who did not follow a recommendation vaccination schedule, or who received heterologous inactivated vaccines during study period were excluded from the study.
Replication	Replication of the analysing was performed using different ways of estimating vaccine effectiveness, as well as a negative binomial regression. All analysis objective at replication were successful.
Randomization	This is an observational study, randomization is not applicable.
Blinding	This is an observational study, blinding is not applicable.

Reporting for specific materials, systems and methods

We require information from authors about some types of materials, experimental systems and methods used in many studies. Here, indicate whether each material, system or method listed is relevant to your study. If you are not sure if a list item applies to your research, read the appropriate section before selecting a response.

Materials & experimental systems

n/a	Involved in the study	
☒	☐	Antibodies
☒	☐	Eukaryotic cell lines
☒	☐	Palaeontology and archaeology
☒	☐	Animals and other organisms
☒	☐	Clinical data
☒	☐	Dual use research of concern

Methods

n/a	Involved in the study	
☒	☐	ChIP-seq
☒	☐	Flow cytometry
☒	☐	MRI-based neuroimaging
